# Net fluorine atom transfer from the perspective of a PCET chemist

**DOI:** 10.1039/d6cc00905k

**Published:** 2026-09-25

**Authors:** Silène Engbers, Johannes E. M. N. Klein

**Affiliations:** a Molecular Inorganic Chemistry, Stratingh Institute for Chemistry, Faculty of Science and Engineering, University of Groningen, Nijenborgh 3 9747 AG Groningen The Netherlands j.e.m.n.klein@rug.nl

## Abstract

Proton coupled electron transfer (PCET) reactions, which encompass hydrogen atom transfer (HAT), concerted proton coupled electron transfer (cPCET) and stepwise variations, have been studied in great detail. This has facilitated the thorough understanding of relevant enzymatic transformations and the efficient development of synthetic strategies. Despite the clear similarities between halogen atom transfer (XAT) and HAT, the conceptual framework used within the PCET community has not yet been extended to radical halogen reactivities. In light of a recent report showing that net homolytic C–F bond cleavage can proceed through a concerted electron fluoride transfer (cEFT) mechanism, we wish to encourage the adoption of a systematic framework for the analysis of radical halogen reactivity, similar to that employed for PCET reactions.

## Abstract 2

Radical halogen reactivity bears similarities to proton coupled electron transfer (PCET). This Mini Review outlines the similarities and differences, encouraging the adoption of conceptual frameworks similar to those developed for PCET.

## Graphical abstract

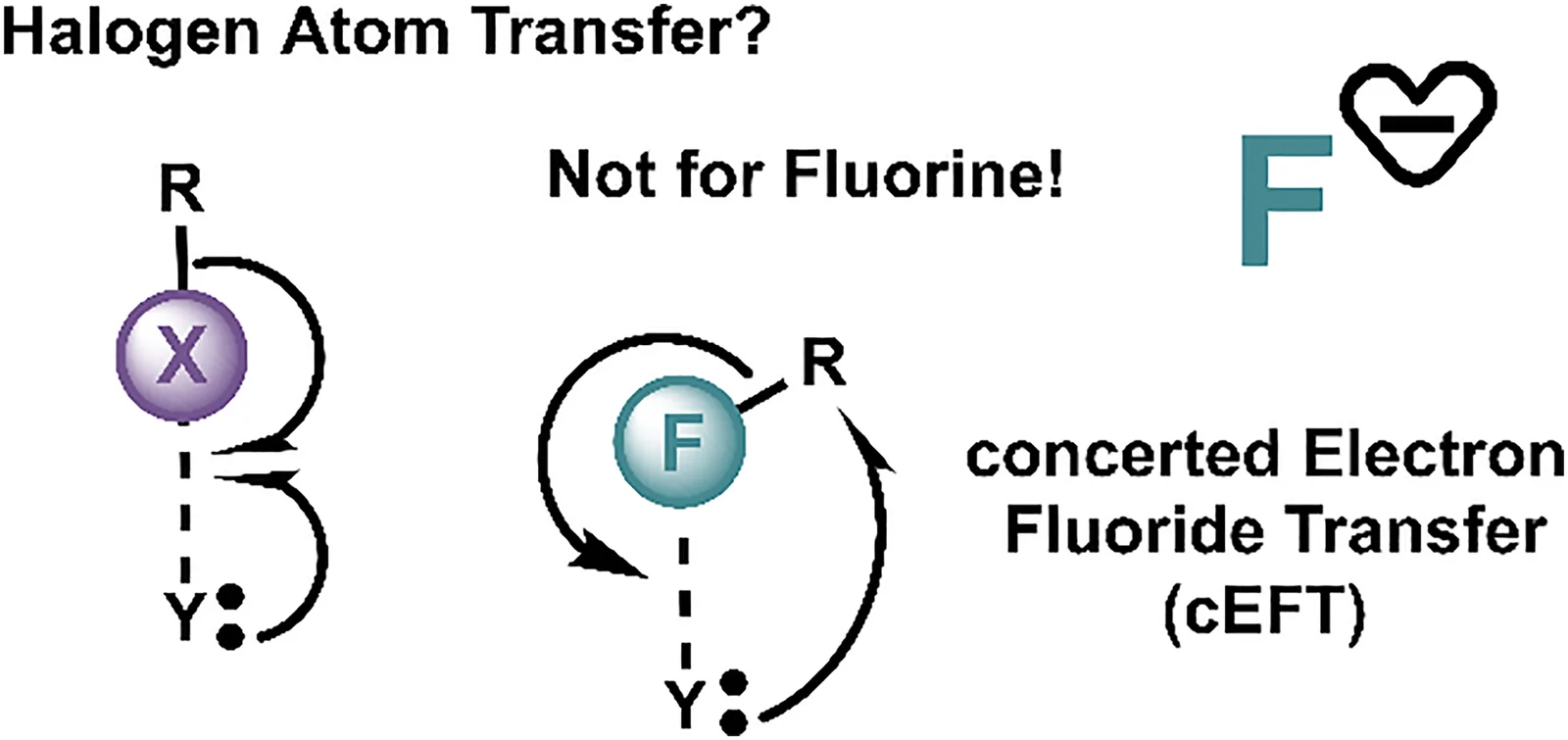

## Introduction: mechanistic considerations for the movement of electrons and cations

The coupled movement of electrons and cations is central to charge transport mechanisms and redox catalysis in biology.^1–3^ Typically, this involves the movement of two electrons and one proton (hydride transfer) or one electron and one proton (proton coupled electron transfer, abbreviated to PCET).^4^ PCET is a broad term that encompasses many mechanistic scenarios that have been rigorously studied both computationally and experimentally.^5–12^ Theoretical^2,4,13–18^ and synthetic reaction^19–21^ chemists have slightly different approaches to understanding these elemental steps, which has consequently led to different perspectives and the application of somewhat different terminologies. An important commonality shared between these perspectives is the treatment of the electron and proton as separate entities. It is in the description of their coupled motion that frameworks can differ slightly.

**Fig. 1:**
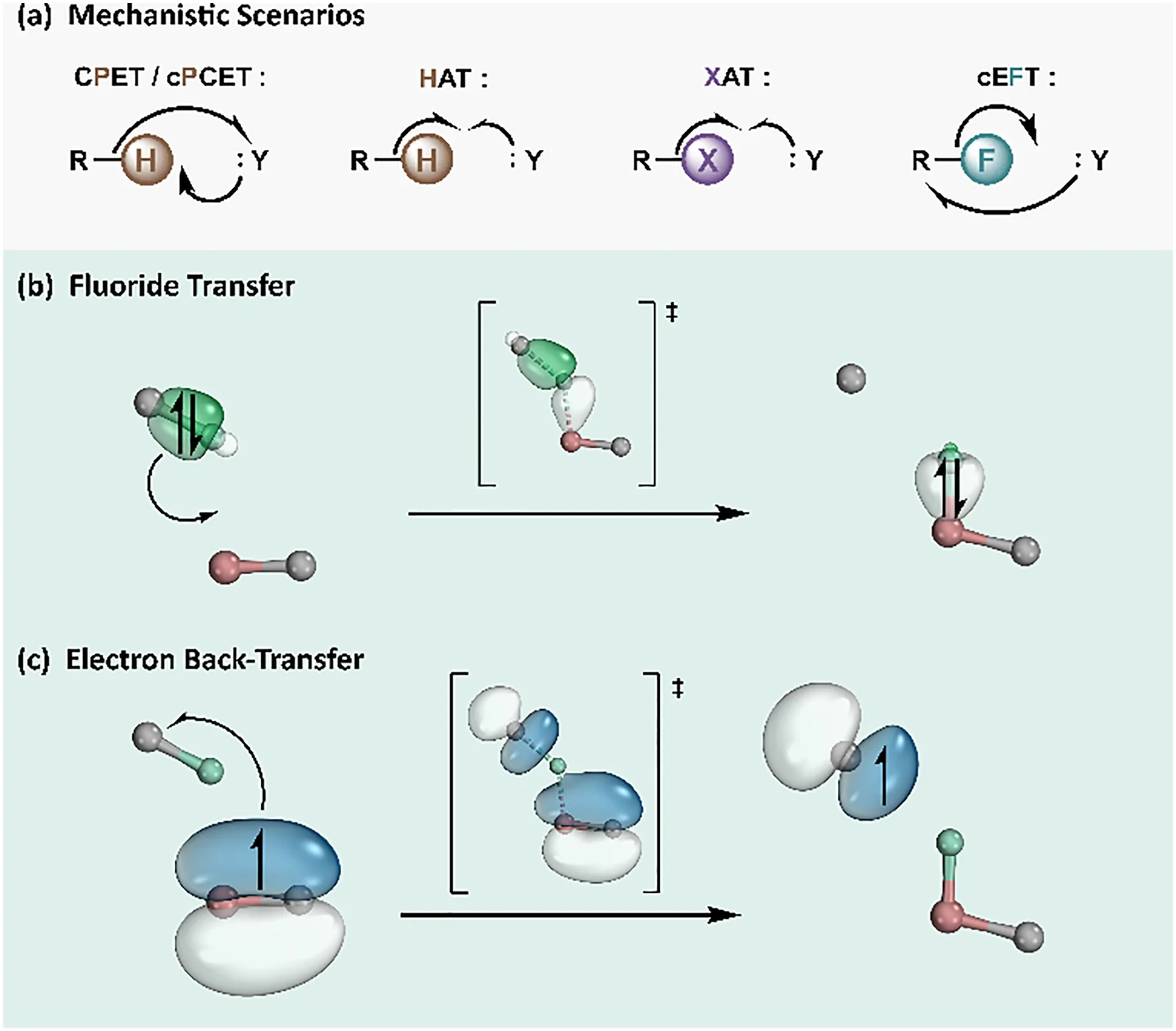
(a) Curly arrow mechanisms for hydrogen atom transfer (HAT), concerted proton coupled electron transfer (cPCET), halogen atom transfer (XAT) and concerted electron fluoride transfer (cEFT). X represents a halogen. Y represents a relevant hydrogen or halogen acceptor molecule. (b) and (c) show the IBO depictions the components of cEFT, (b) fluoride transfer and (c) electron back-transfer. Fluoride is shown in green, carbon in grey, and boron in pink.

Some inorganic computational chemists (including ourselves) have taken a rather simplistic view on the topic wherein electron flow diagrams, often based on intrinsic bond orbital (IBO) changes along the reaction coordinate,^22–24^ are utilised to differentiate between different mechanistic scenarios. In particular, two types of concerted PCET have been explored (Fig. 1a):^24–28^ hydrogen atom transfer (HAT), where the proton and electron move together as one discrete hydrogen atom so that the electron becomes part of the newly formed Y–H bond, or concerted proton coupled electron transfer (cPCET, also referred to as CPET), where the electron and proton are transferred along different pathways so that the electron is embedded elsewhere in the acceptor molecule. Both mechanisms can be either synchronous, wherein the electron and proton travel simultaneously, or asynchronous, wherein the electron and proton are transferred within the same step but with disconnect along the reaction coordinate.

Although initial interest in PCET was particularly driven by understanding biological processes, it has also gained an important role in synthetic chemistry as it provides elegant access to one-electron reaction steps.^29–34^ Synthetic chemists have more recently extended this approach to one-electron chemistry by exploiting homolytic cleavage of organohalide bonds, and have named it halogen atom transfer (XAT).^35–37^ Notably, radical halogen reactivity also occurs in nature, see for example the mechanism of halogenases (*vide infra*).^38–40^

The concept of coupling electron and proton motion is theoretically similar of coupling the motion of an electron and a cationic halogen. However, radical halogen reactivity has rarely been investigated using the tools developed for PCET. Due to the larger electronegativity of halogens compared to protons,^41^ it may have been assumed that XAT (analogous to HAT, Fig. 1a) is essentially always operative. Beyond considerations of asynchronicity (also referred to as polarised XAT),^35^ it seemed unlikely that rigorous mechanistic analysis would provide useful insight.

Although our group has recently been interested in generating and utilising electrophilic chlorine,^42–45^ when it comes to radical halogen reactivity fluorine is particularly interesting. In the context of radical halogen reactivity, fluorine is generally treated separately.^46–54^ C–F bonds are remarkably strong (about 110 kcal mol^−1^ for an aliphatic C–F bond)^55^ and are as such thermodynamically challenging to cleave.^46,47,50,53,56–58^ Additionally, a large kinetic barrier is associated with homolysis of a C–F bond, resulting from the exceptional electronegativity of fluorine.^59^ Promoting radical fluorine reactivity has thus been a tremendous challenge, but one with high reward. The unique chemical properties of fluorine have made it ubiquitous in the design of new pharmaceuticals.^60^ The development of efficient routes to (late-stage)^61^ C–F bond formation^62,63^ and selective C–F bond cleavage from polyfluorinated compounds^64,65^ is thus highly beneficial to society.

To facilitate reaction design, the Xue group recently investigated the homolytic C–F bond cleavage computationally and compared it to other halogens.^66^ We were particularly interested in their use of IBO changes along the reaction coordinate (as commonly done for PCET) to identify a new mechanistic scenario for radical fluorine reactivity. We will highlight their findings and further tie this to experimental observations of fluorine atom abstraction and formation (which together comprise net radical fluorine reactivity). We do not intend this Mini Review to be a comprehensive overview of the literature. Instead, we aim to encourage the adoption of a conceptual framework for radical halogen reactivity that bears similarities to that used for PCET.

## Concerted electron fluoride transfer (cEFT)

**Scheme 1:**
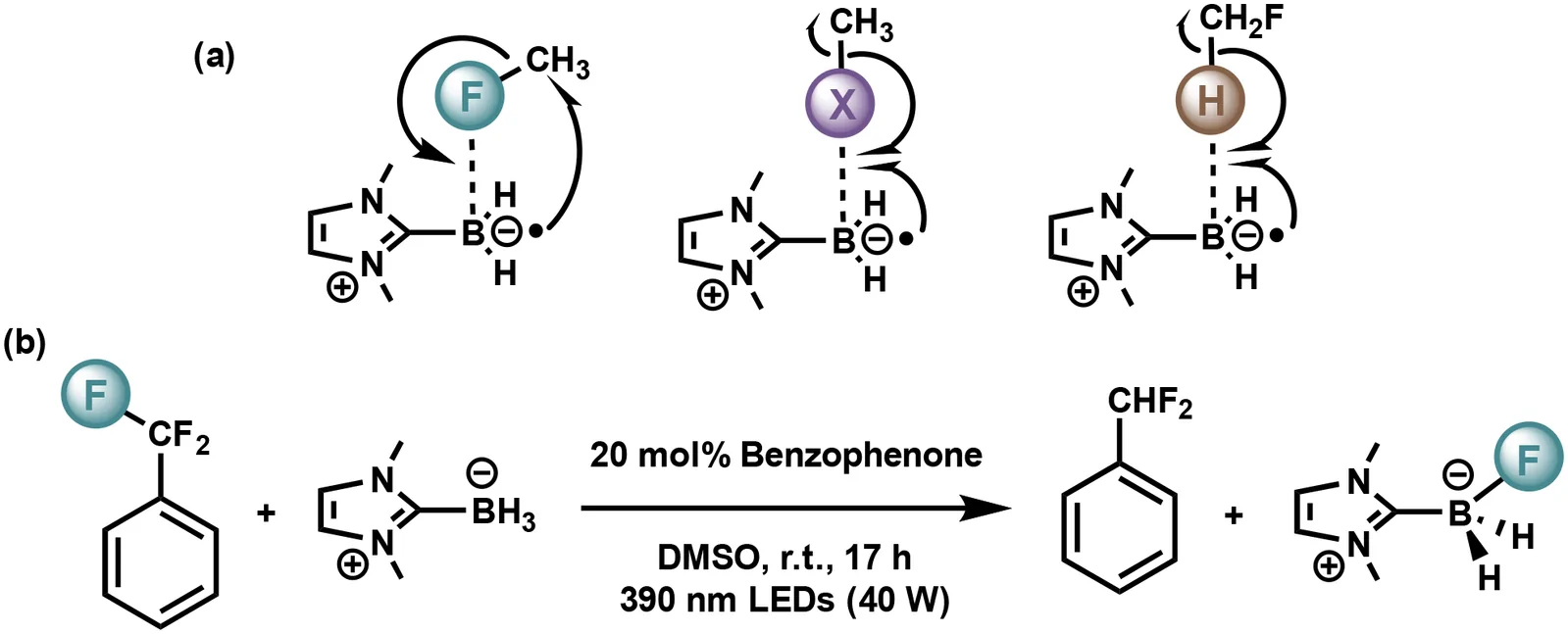
(a) Mechanistic divergence of fluorine in halogen/hydrogen atom abstraction by a Lewis base-boryl radical as reported by the Xue group.^66^ X represents Cl, Br, or I; (b) experimentally observed fluorine atom abstraction by a Lewis base-boryl system reported by the Lee group.^67^

The mechanism of fluorine atom abstraction from fluoroalkanes by Lewis base-boryl radicals, such as *N*-heterocyclic carbene-boryl radicals, was recently inspected computationally by the Xue group.^66^ Analysis of the IBO changes along the reaction coordinate suggest that XAT is not operative. Instead, a fluoride atom is transferred to the boron and the unpaired electron on the boron is simultaneously back-transferred to the fluoroalkane substrate (Scheme 1a, Fig. 1b and c). The electron back-transfer provides a net fluorine atom abstraction despite the transfer of a cationic fluorine atom coupled to two electrons as one entity (fluoride). This mechanism is thought to be operative for a range of Lewis base-boryl radicals and the authors termed this mechanistic scenario concerted electron fluoride transfer (cEFT), Fig. 1.^66^ cEFT resembles CPET/cPCET in some ways, but instead of a positive and negative charge travelling in the same direction, it entails two negative charges travelling in opposite directions.

The Xue group further investigated the abstraction of other halogens and hydrogen by the same system.^66^ For fluorine, at the transition state, an obtuse angle of 128.9 ° was computed, whereas linear geometries are found for all other halogens and hydrogen. The well-known XAT and HAT mechanisms are operative for all halogens (except fluorine) and hydrogen, respectively (Scheme 1a).^66^ While in these examples a mechanistic difference is observed based on bond angles, it remains to be seen whether this is of general validity. This divergence in mechanistic profiles highlights the uniqueness of fluorine, for which its remarkably large electronegativity^41^ enforces the transfer of a fluoride atom.

Almost simultaneously to this computational report, the experimental equivalent was published by the Lee group (Scheme 1b), exemplifying the experimental relevance of the cEFT mechanism.^67^ The Lewis base-boryl radical in this system is thought to be generated *in situ* by HAT with the help of a photo-activated radical initiator.

## How relevant and general is cEFT?

**Scheme 2:**
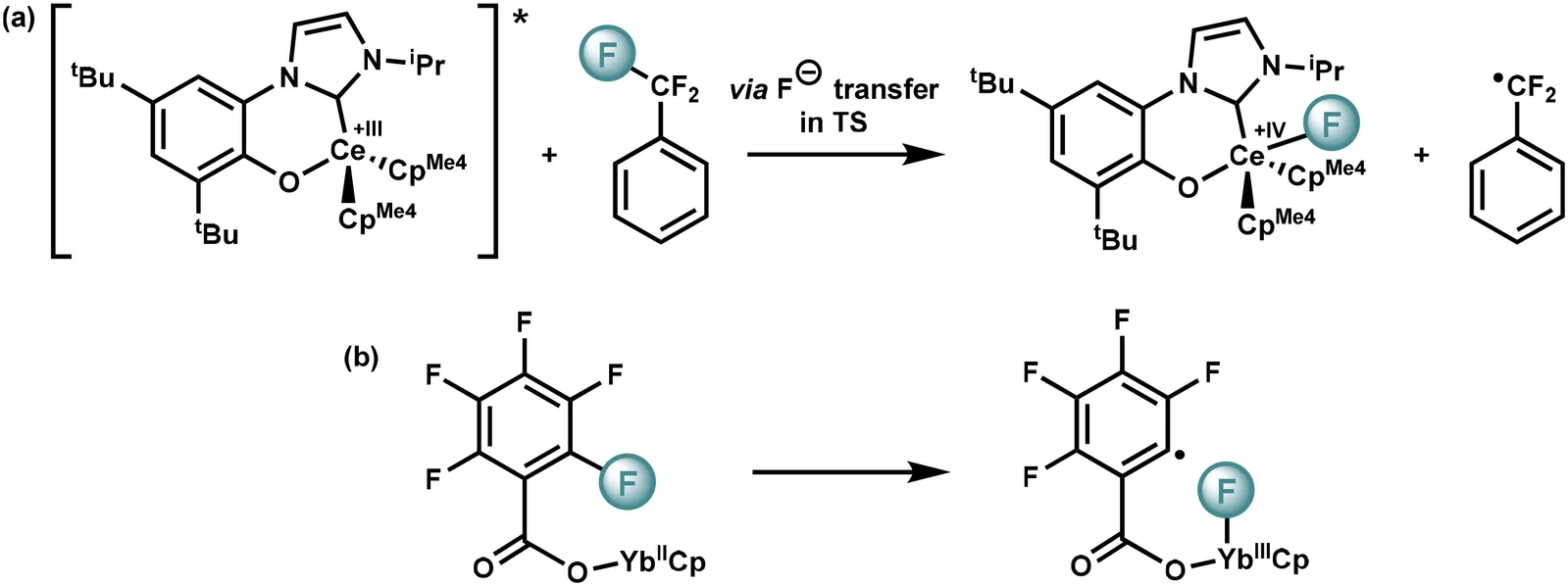
Possible cEFT reported (a) by the groups of Maron and Arnold,^68^ and (b) the Deacon group.^69–71^ Cp stands for cyclopentadienyl and Cp^Me4^ stands for tetra(methyl)cyclopentadienyl. The * indicates an excited state molecule.

Although the term cEFT was only recently introduced,^66^ similar reactivities have been reported in the past, suggesting that the Xue group's findings were not coincidental. For example, defluorination of C(sp^3^)–F bonds by lanthanide photocatalysts was reported by the Maron and Arnold groups a few years ago.^68^ Herein, an excited state Ce or La complex performs fluorine atom abstraction (Scheme 2a). Computational mechanistic investigations indicate the transfer of a fluoride ion in the transition state, which results in the formation of a carbon-centered radical. For this to be possible, electron back-transfer must be operative, making it likely that cEFT is happening here. Similarly, cEFT may be operative in reported Yb-mediated fluorine atom abstractions (Scheme 2b), as the authors suggest that intramolecular electron transfer and fluoride abstraction occur simultaneously in a six-membered cyclic transition state.^69–71^

**Scheme 3:**
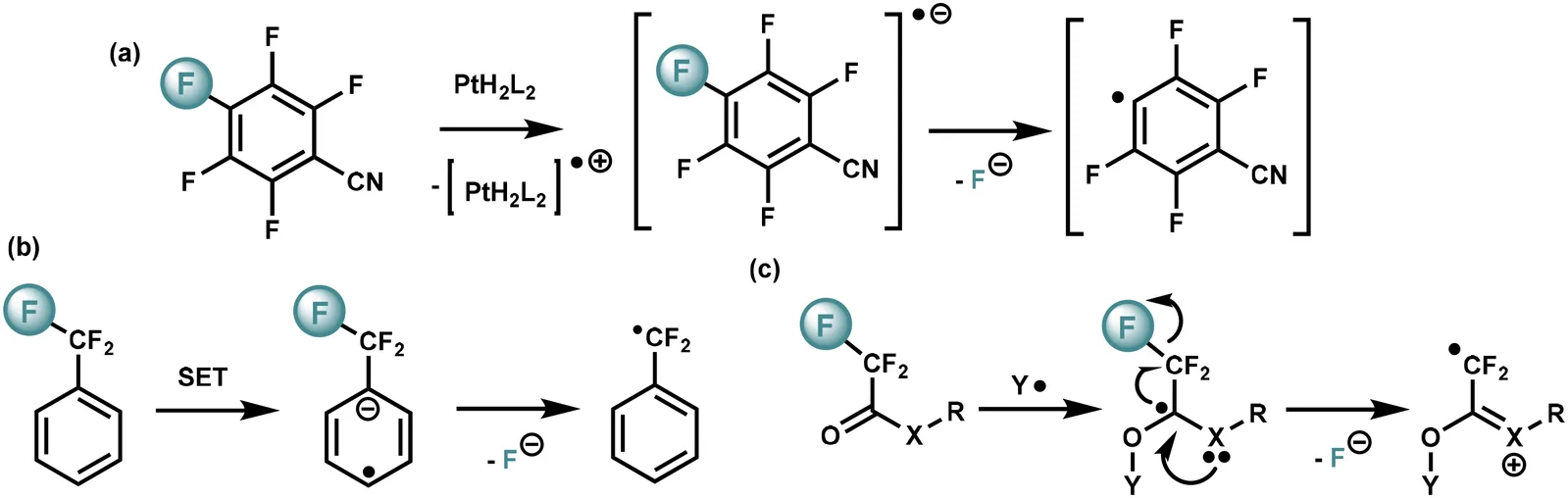
(a) Stepwise fluorine atom abstraction by a Pt complex, L = PCy_3_, as reported by the Rüegger group;^72^ (b) an example of stepwise fluorine atom abstraction by single electron transfer (SET) from a photo-activated photocatalyst, as reported by the Jui group;^74^ (c) effective halogen atom abstraction over two steps by use of a spin center shift as reported by the groups of Wang and Houk.^76^ An example of Y is a 4-dimethylaminopyridine-boryl radical and X is often an amine.

Stepwise variations of net homolytic C–F bond cleavage that relate to the term cEFT have been observed in several examples. Although stepwise PCET has been reported for both electron transfer preceding proton transfer and *vice versa*,^3^ for fluorine the electron back-transfer typically precedes fluoride abstraction.^47^ This also often leads to release of fluoride to solution rather than the formation of a new X–F covalent bond. Select examples of such a scenario include the defluorination of fluorobenzenes by Pt or Ru hydrides (Scheme 3a),^72,73^ or the defluorination of trifluoromethyl arenes by (organic) photocatalysts (Scheme 3b).^74,75^ The groups of Wang and Houk have also recently applied spin center shifts to achieve C(sp^3^)–F defluorination (Scheme 3c).^76,77^ Such a mechanism could be considered similar to stepwise cEFT.

**Scheme 4:**
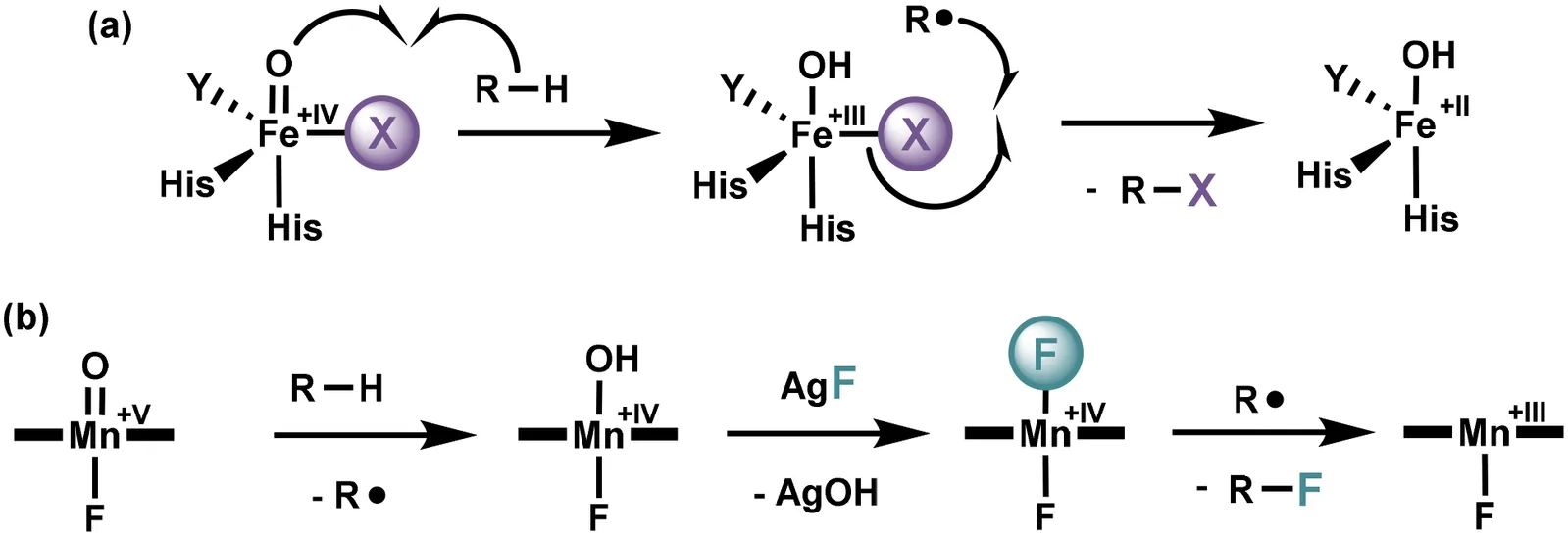
(a) Relevant steps in the mechanism of halogenases, (b) the fluorination method developed by the Groves group.^95–98^ The thick horizontal line represents either a porphyrin or salen ligand.

Inspecting the mechanism of the formation of C–F bonds, which we may view as the microscopic reverse of fluorine atom abstraction, can provide further insights. Carbon–halogen bond formation through radical intermediates is prominent in nature for chlorine and bromine and is performed by halogenases.^38–40^ These achieve hydrogen atom abstraction by an oxoiron(IV) species followed by radical rebound with the iron-bound halogen to form the new C–X bond (Scheme 4a).^78^ Out of the halogens, fluorine-containing organic molecules are particularly rare in nature,^55,79–81^ and the only known fluorinating enzymes proceed through a nucleophilic pathway.^82^ Considering the particularly large oxidation potential of fluorine, it is unlikely that enzymes exist that have evolved to perform fluorination *via* oxidative pathways.^39,81^

Because of nature's scarce use of fluorine, the synthetic selective fluorination of organic molecules is of high interest for the production of, for example, pharmaceutically relevant products.^62,83–86^ Synthetic chemists have developed numerous methods to achieve this, including both oxidative and radical pathways.^62,87–94^ Halogenase-like reactivity involving radical rebound of a carbon-based radical was developed by the Groves group (Scheme 4b).^95–98^ Herein, manganese porphyrin or salen complexes are used, which can attain higher oxidation states than in halogenase enzymes. DFT calculations confirm the presence of a viable transition state involving transfer of a net fluorine atom.^95,97^ Although it is unclear from currently published data whether XAT or cEFT is operative, it is noteworthy that reported transition states exhibit C–F–Mn bond angles of 170 to 178°.^95,97^

**Scheme 5:**
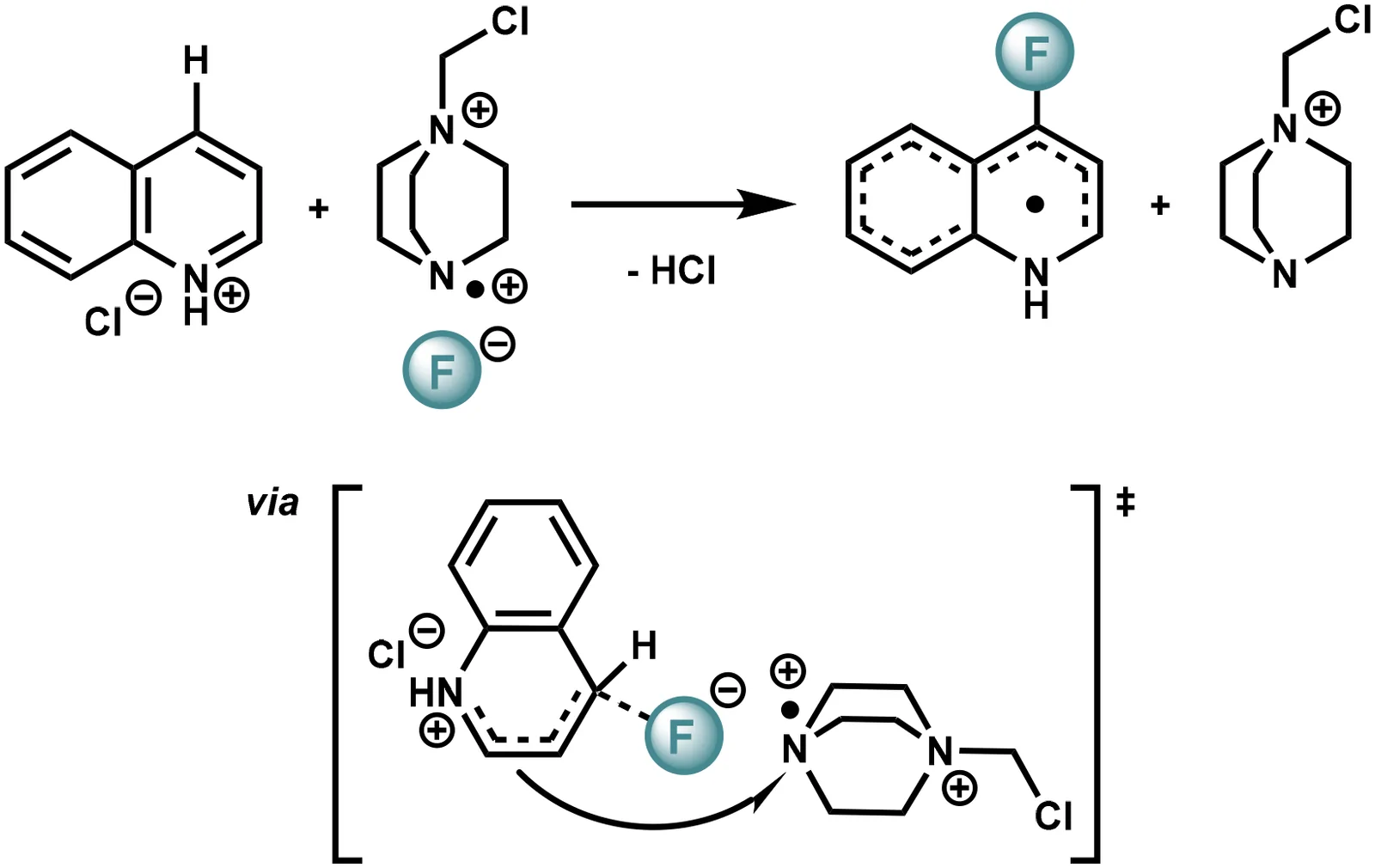
cEFT reported by the Ritter group.^99^

A particularly relevant example in the context of cEFT related to the formation of C–F bonds is the electron-transfer enabled concerted nucleophilic fluorination of quinolines reported by the Ritter group (Scheme 5).^99^ Herein, it is postulated that fluoride transfer and electron back-transfer occur in a single transition state, followed by barrier-less deprotonation. This concerted mechanism is promoted by employing protonated quinoline, which lowers the barrier to fluoride attack, together with a one-electron oxidised fluoride source to facilitate the electron back-transfer. The reaction design considerations presented by the Ritter group show how cEFT can be promoted and as such utilised to facilitate challenging transformations.

## Discussion

In the examples above, our tentative mechanistic assignment is based on the limited information reported in each case. For certain examples, such as the Mn-catalysed fluorination reported by the Groves group, the operative mechanism is still quite unclear and revisiting (computational) investigations into the mechanism would be quite illuminating. For this, we would recommend analysing the reaction in a similar manner to a PCET reaction, for example by performing an IBO analysis over the course of the reaction as done by the Xue group for the reaction Lewis base-boryl radicals with fluoroalkyls (*vide supra*).^66^

For future studies, routinely identifying and reporting the mechanistic label (XAT or cEFT) for new radical fluorine reactivity will provide a clear overview of accessible fluorine reactivity. The design requirements for an XAT and cEFT reaction are unlikely to be similar, and the choice of reaction partner will have a significant influence. Reported data often hints that cEFT is operative, but gives us no indication on where the boundary with XAT may lie. The implementation of an analytic approach like that used for PCET will help provide a clearer understanding of the scope and boundary cases for XAT and cEFT. This will enable rational design of radical fluorination and expand our chemical toolbox.

Although we have only highlighted cases of radical fluorine reactivity here, one may ask themselves if under certain circumstances chlorine might be able to undergo a cEFT mechanism.

## Conclusion

The exceptional electronegativity of fluorine facilitates unique reactivity for this halogen in halogen atom abstractions. Instead of the typical XAT mechanism, fluorine can undergo cEFT, wherein a cationic fluorine atom and to two electrons travel as one unit (fluoride), coupled with electron back-transfer to provide net radical reactivity. Although we have highlighted some examples of cEFT as a concerted mechanism as well as step wise variations, it is equally possible that XAT could be operative in some cases. Moving forward, we recommend that radical fluorine reactivity be viewed within a similar framework to PCET. Uniform application of the cEFT nomenclature and its distinction from XAT will aid in recognising such patterns and provide a clearer understanding on how to design reaction conditions to facilitate challenging radical transformations involving fluorine.

## Conflicts of interest

There are no conflicts to declare.

## Data Availability

No primary research data are included in this review article.
